# Culture-Based Microbiological Assessment of Dentin Biomaterials Prepared from Long-Term Refrigerated Human Teeth: A Pilot Study Toward Evidence-Based Tooth Banking

**DOI:** 10.3390/jfb17080388

**Published:** 2026-08-05

**Authors:** Robert Dłucik, Anna Mertas, Zenon P. Czuba, Karolina Ziaja, Bogusława Orzechowska-Wylęgała

**Affiliations:** 1 Dłucik Dental Clinic, 40-737 Katowice, Poland; 2 Children’s Maxillofacial Surgery Clinic, Chair of Pediatric Surgery, Medical University of Silesia, 40-752 Katowice, Poland; borzechowska-wylegala@sum.edu.pl; 3 Department of Microbiology and Immunology, Faculty of Medical Sciences in Zabrze, Medical University of Silesia, 40-055 Katowice, Poland; amertas@sum.edu.pl (A.M.); zczuba@sum.edu.pl (Z.P.C.)

**Keywords:** tooth banking, Evidence-Based Tooth Banking (EBTB), dentin-derived biomaterials, autogenous dentin graft, long-term refrigerated storage, culture-based microbiological assessment, MALDI-TOF MS, bone regeneration

## Abstract

Dentin derived from extracted human teeth has emerged as a promising autogenous biomaterial for bone regeneration owing to its structural and biological similarity to bone tissue. Growing clinical interest in dentin-derived graft materials has also stimulated the development of tooth-banking concepts, in which extracted teeth are preserved for future regenerative applications. However, microbiological contamination remains one of the principal concerns associated with long-term storage before clinical use. This in vitro study evaluated the microbiological status of dentin biomaterials prepared from human teeth stored under long-term refrigerated conditions (approximately 4 °C) and subsequently processed using three commercially available dentin-processing systems: BonMaker (BM), Tooth Transformer (TT), and Smart Dentin Grinder (SDG). A total of 72 extracted teeth collected between 2018 and 2025 were allocated to experimental groups and processed into 24 pooled dentin samples. Five freshly extracted teeth served as controls. Culture-based microbiological assessment was performed before and after dentin processing using Schaedler Broth K3 culture media under aerobic and anaerobic conditions. Microorganisms isolated from positive cultures were identified by matrix-assisted laser desorption/ionization time-of-flight mass spectrometry (MALDI-TOF MS). Baseline microbiological assessment detected bacterial contamination in 2 of 24 pooled dentin samples (8.3%) and in 4 of 5 control teeth (80.0%). The identified microorganisms included *Staphylococcus epidermidis*, *Actinomyces viscosus*, *Streptococcus anginosus*, *Streptococcus mutans*, and *Staphylococcus hominis*. No microbiologically detectable bacterial growth by culture-based methods was observed following processing with BM, TT, or SDG, irrespective of storage duration, including teeth preserved under refrigerated conditions for up to seven years. Within the limitations of this culture-based investigation, the evaluated dentin-processing protocols effectively eliminated microbiologically detectable cultivable bacterial contamination from long-term refrigerated dentin samples. These findings provide preliminary culture-based microbiological evidence supporting the feasibility of long-term refrigerated tooth preservation under the investigated storage conditions as one component of future tooth-banking research. Since culture-based microbiological methods do not detect viable but non-culturable microorganisms or other non-cultivable pathogens, the present findings should not be interpreted as evidence of complete sterility, overall biological safety, or the superiority of refrigerated storage over alternative preservation methods. Further studies employing molecular microbiological techniques together with biological and physicochemical analyses are required to comprehensively validate long-term tooth-banking protocols.

## 1. Introduction

The search for biomaterials with high biocompatibility and regenerative potential remains a major focus in regenerative medicine and dentistry. In oral surgery and implantology, bone augmentation procedures are frequently required to restore alveolar bone defects and facilitate implant placement. Among the available grafting materials, autogenous tissues remain the gold standard owing to their excellent biological integration, absence of immunogenic reactions, and favorable remodeling characteristics [1,2,3].

In recent years, increasing attention has been directed toward dentin derived from extracted human teeth as a promising autogenous biomaterial for bone regeneration. Human dentin exhibits substantial chemical and structural similarity to bone tissue and consists primarily of hydroxyapatite, type I collagen, and numerous non-collagenous proteins, including bone morphogenetic proteins (BMPs), transforming growth factor-β (TGF-β), and insulin-like growth factors (IGFs), which contribute to its osteoconductive and osteoinductive properties [4,5,6,7,8]. Numerous experimental and clinical studies have demonstrated predictable regenerative outcomes following the use of dentin-derived biomaterials in alveolar ridge preservation, sinus floor augmentation, and implant-related bone regeneration [9,10,11]. Beyond their regenerative potential, dentin-derived biomaterials represent a unique example of patient-specific biological recycling, transforming extracted teeth from biological waste into valuable autogenous graft materials.

The growing clinical use of dentin-derived grafts has stimulated interest in the concept of tooth banking as a potential future approach to preserving patient-specific biological resources for regenerative applications. Early studies demonstrated the feasibility of preserving extracted teeth for future clinical use, whereas subsequent investigations proposed organized tooth-banking systems capable of supporting personalized regenerative therapies [10,11,12,13].

Although immediate chairside preparation of dentin grafts has become increasingly common, delayed regenerative treatment often requires extracted teeth to be stored for months or even years before clinical reuse. Consequently, long-term preservation represents one of the principal scientific and practical challenges in the development of standardized tooth-banking protocols.

Unlike superficial contamination, microorganisms may penetrate deeply into dentinal tubules, where they are protected within the dentin microstructure and may survive conventional surface decontamination procedures [14,15,16,17,18,19]. Following mechanical grinding, these bacteria may become exposed within dentin particles intended for clinical application. Therefore, microbiological assessment of dentin-derived biomaterials should consider not only contamination of the external tooth surface but also microorganisms residing within the dentinal tubule system [14,15,16,17,18,19].

To facilitate the clinical use of autogenous dentin, several commercially available chairside processing systems have been developed to convert extracted teeth into particulate dentin graft materials. Among the most widely used are BonMaker (BM; Korea Dental Solution Co., Ltd., Busan, Republic of Korea), Tooth Transformer (TT; Tooth Transformer S.r.l., Milan, Italy), and Smart Dentin Grinder (SDG; KometaBio Inc., Tenafly, NJ, USA) [20,21,22]. Although these systems differ in their grinding mechanisms, chemical decontamination protocols, and partial demineralization procedures, all are intended to produce clinically applicable dentin-derived biomaterials suitable for regenerative applications [23,24,25]. However, relatively little is known about their microbiological performance when teeth undergo prolonged storage before processing.

In routine clinical practice, refrigerated storage at approximately 4 °C represents one of the most accessible preservation methods because it is inexpensive, readily available, and capable of limiting bacterial proliferation compared with storage at ambient temperature. Nevertheless, refrigeration should not be regarded as the optimal preservation strategy. At present, insufficient evidence exists to determine the relative advantages and limitations of refrigerated storage compared with freezing, cryopreservation, lyophilization, or other preservation methods for extracted teeth intended for regenerative applications [12,13,22,26,27,28].

The present study was designed as a pilot investigation focusing exclusively on culture-based microbiological assessment. Its primary objective was to determine whether microbiologically detectable bacterial contamination persisted following prolonged refrigerated storage and standardized processing using three commercially available dentin-processing systems. Accordingly, the findings should be interpreted solely within the scope of conventional culture-based microbiological methods and should not be considered evidence of complete sterility, overall biological safety, or the clinical suitability of long-term stored dentin-derived biomaterials. Furthermore, the present investigation was not designed to compare different tooth preservation strategies and therefore does not permit conclusions regarding the superiority of refrigerated storage over alternative preservation methods.

We hypothesized that standardized dentin-processing protocols would eliminate microbiologically detectable cultivable bacterial contamination irrespective of storage duration. Therefore, the aim of this study was to perform a culture-based microbiological assessment of dentin biomaterials prepared from human teeth stored under long-term refrigerated conditions and subsequently processed using the BM, TT, and SDG systems. The findings are intended to provide preliminary microbiological evidence that may contribute to the future multidisciplinary scientific validation of tooth banking rather than to establish a clinical or regulatory standard for long-term tooth preservation.

## 2. Materials and Methods

### 2.1. Study Design

This pilot in vitro study was designed as a descriptive culture-based microbiological investigation of dentin-derived biomaterials prepared from human teeth stored under long-term refrigerated conditions. Aerobic and anaerobic microorganisms were assessed before and after dentin processing using three commercially available dentin-processing systems (BM, TT, and SDG).

The investigation focused exclusively on culture-based microbiological assessment and was intended to determine whether microbiologically detectable bacterial contamination persisted following prolonged refrigerated storage and standardized dentin processing. Biological activity, physicochemical stability, inflammatory response, and clinical performance were beyond the scope of the present study. Accordingly, the methodology employed in the present study does not permit conclusions regarding complete sterility, overall biological safety, clinical applicability, or the superiority of refrigerated storage over alternative preservation methods.

**Figure 1:**
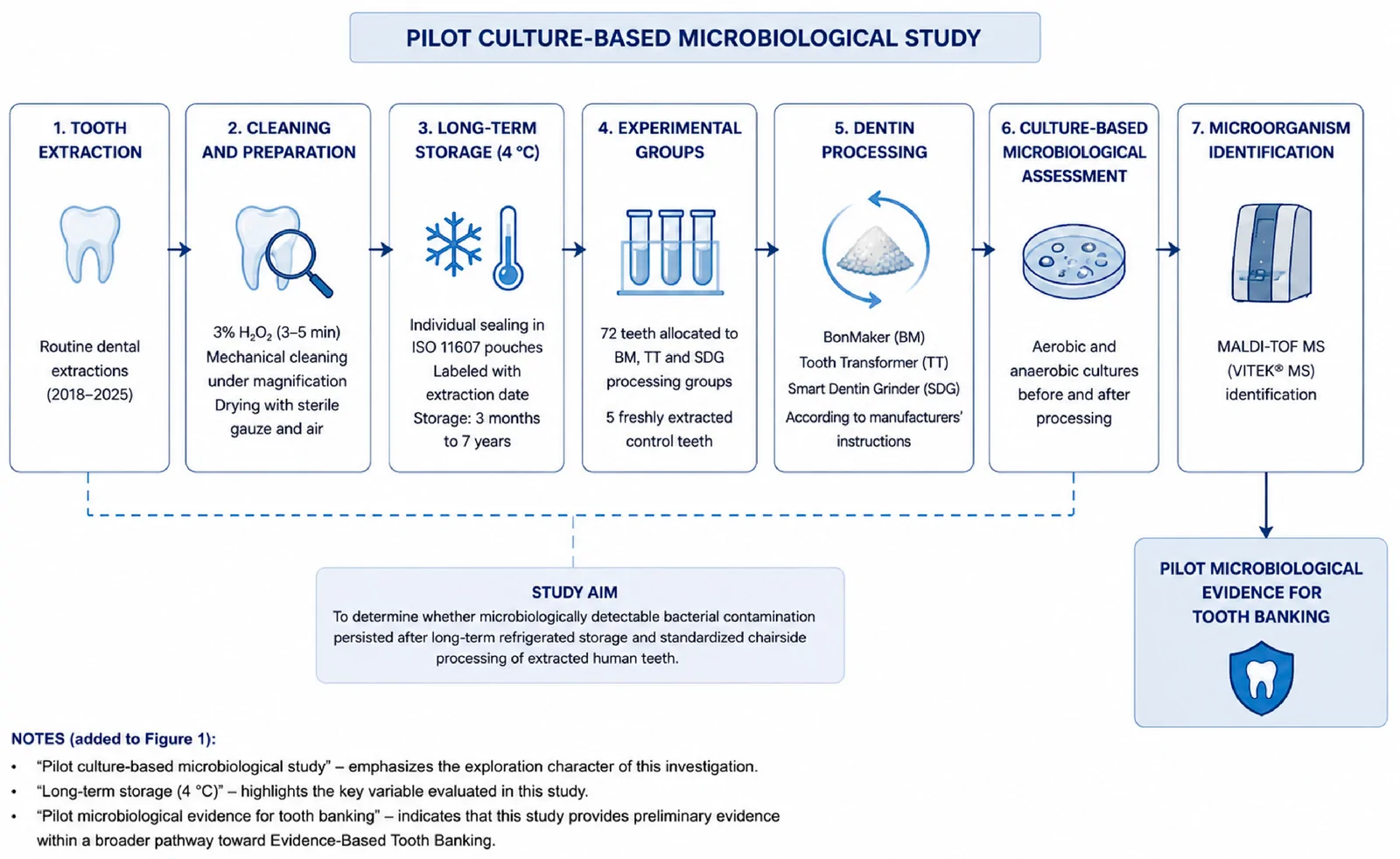
Experimental workflow of the pilot culture-based microbiological study.

The study was conducted in 2025 at two centers. Sample preparation and dentin processing were performed at Dłucik Dental Clinic (Katowice, Poland), whereas microbiological analyses were conducted at the Department of Microbiology and Immunology, Medical University of Silesia in Katowice, Poland. The study workflow is presented in Figure 1.

### 2.2. Tooth Collection, Preparation and Storage

Human teeth extracted between 2018 and 2025 during routine dental treatment at Dłucik Dental Clinic (Katowice, Poland) were collected and preserved for subsequent preparation of dentin-derived biomaterials. The study included teeth extracted for routine clinical indications. Because the teeth had been collected over several years as part of routine clinical practice, detailed information regarding the indication for extraction (e.g., caries, periodontal disease, orthodontic or prosthetic reasons) was not consistently available for all specimens and therefore was not included as an experimental variable. This limitation is acknowledged in the Discussion.

**Figure 2:**
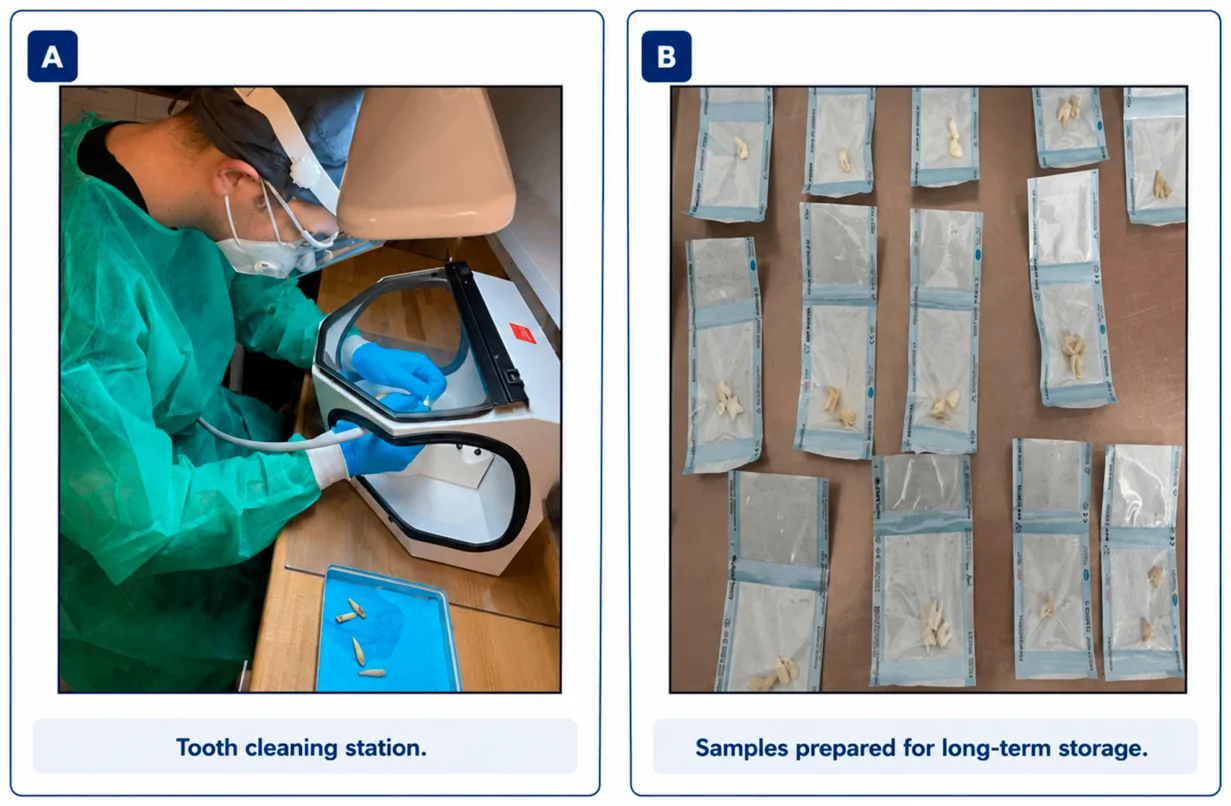
Cleaning of extracted teeth (**A**) and representative samples prepared for long-term refrigerated storage (**B**).

Immediately after extraction, residual soft tissues, dental calculus, restorative materials, and visible carious lesions were mechanically removed using diamond burs under ×3.5 magnification. The teeth were subsequently immersed in 3% hydrogen peroxide (H_2_O_2_) for 3–5 min, rinsed with sterile distilled water, dried with sterile gauze and compressed air, individually packaged in sterile self-sealing sterilization pouches compliant with ISO 11607, and labeled with the extraction date (Figure 2).

Storage was performed in a dedicated medical refrigerator maintained at approximately 4 °C according to the manufacturer’s specifications. Teeth remained individually packaged throughout the storage period, ensuring complete sample identification and traceability until allocation to the experimental groups. Refrigerated storage represented the routine preservation protocol historically used in our institution during the study period. The objective of the present investigation was not to compare different storage methods but to evaluate the microbiological status of dentin-derived biomaterials prepared from teeth preserved under routine refrigerated storage conditions. Consequently, no comparisons with freezing, cryopreservation, lyophilization, or room-temperature storage were performed. Accordingly, the present protocol should not be interpreted as supporting refrigerated storage over alternative preservation strategies.

Storage duration ranged from 3 months to 7 years, depending on the year of extraction. No additional decontamination procedures were performed during the storage period prior to the standardized dentin-processing protocols described below.

### 2.3. Experimental Groups

A total of 77 extracted human teeth were included in the study. Seventy-two teeth were allocated to the experimental groups, whereas five freshly extracted teeth served as the control group for baseline microbiological assessment.

The experimental material consisted of teeth stored at 4 °C in a Beko Professional medical refrigerator (Beko, Turkey) for different periods ranging from 3 months to 7 years. To evaluate the potential influence of storage duration, the teeth were divided into eight storage groups according to the year of extraction (2018–2025). Each storage group consisted of nine teeth, which were subsequently assigned to one of three dentin-processing systems: BM, TT, or SDG. Accordingly, each processing subgroup contained three teeth (Table 1).

For microbiological analysis, dentin particles obtained from the three teeth within each processing subgroup were pooled to generate one representative microbiological sample. Pooling was intentionally selected to standardize microbiological processing, ensure sufficient sample volume for culture analyses, and provide a representative assessment of each experimental subgroup. Consequently, the microbiological unit of analysis consisted of 24 pooled dentin samples rather than 72 individual teeth.

The control group consisted of five freshly extracted teeth that underwent the same initial cleaning procedure but were not subjected to prolonged storage or dentin-processing protocols before microbiological evaluation.

The authors acknowledge that pooling precluded identification of the individual tooth responsible for positive microbiological findings and did not permit tooth-level statistical analyses. This limitation has been considered in the interpretation of the results and is further discussed in the Discussion section. Pooling was planned before initiation of the microbiological analyses and was not introduced after completion of the experiment.

### 2.4. Baseline Culture-Based Microbiological Assessment

Prior to dentin processing, the stored teeth were removed from refrigerated storage and transferred to a surgical environment under strictly aseptic conditions. All procedures were performed by two operators (a dentist and a surgical assistant) using sterile gloves, sterile surgical gowns, surgical caps, FFP2 masks, protective face shields, and protective eyewear to minimize the risk of environmental contamination.

The teeth were dried using sterile gauze and compressed air. Mechanical fragmentation was subsequently performed using a sterile surgical mortar and hammer to obtain dentin particles suitable for microbiological assessment.

**Figure 3:**
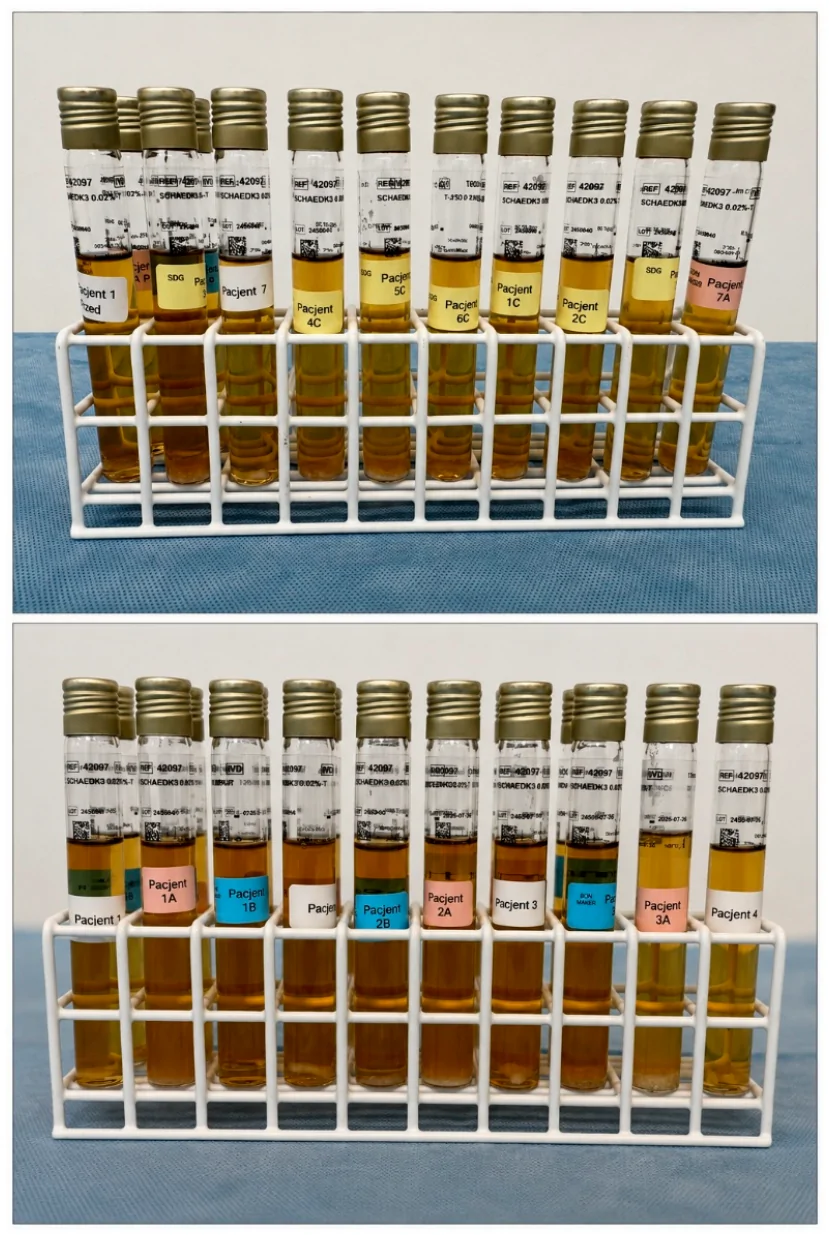
Schaedler Broth K3 culture medium containing dentin fragments immediately before incubation at 37 °C.

Following fragmentation, the pooled dentin particles were thoroughly homogenized, and a representative portion of each pooled sample was aseptically transferred into Schaedler Broth K3 supplemented with vitamin K3 (bioMérieux, Marcy-l’Étoile, France) using a sterile biomaterial spoon (Figure 3). This baseline culture was performed before the application of any chemical decontamination or dentin-processing protocol to evaluate the presence of microbiologically detectable cultivable microorganisms by conventional culture-based methods.

Strict aseptic technique was maintained throughout all microbiological procedures. Culture tubes were opened only for the minimum time required for sample transfer, and all manipulations were performed using sterile instruments under aseptic conditions to minimize environmental contamination. The tube openings were flame sterilized immediately before closure. 

**Figure 4:**
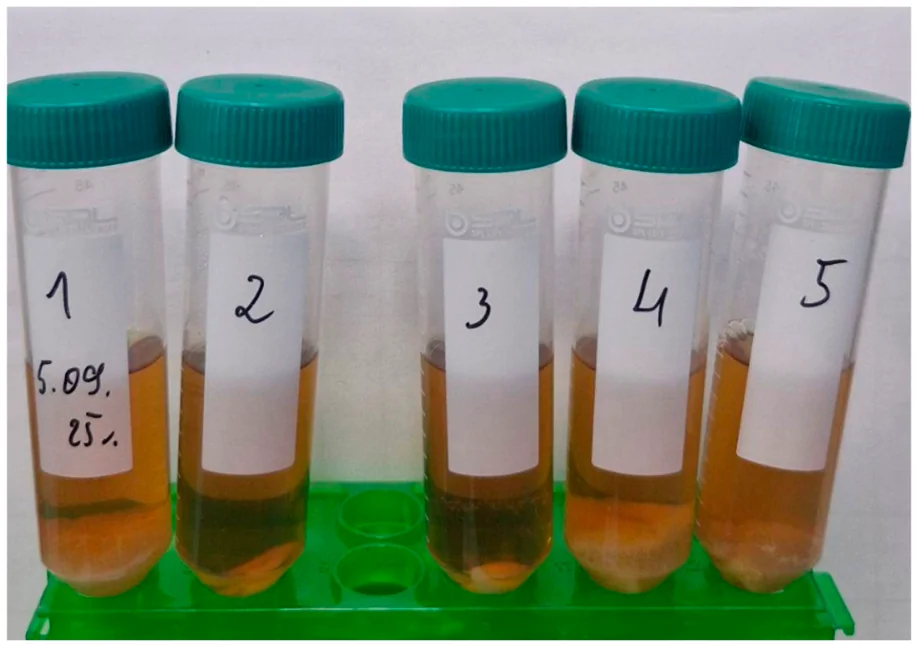
Schaedler Broth K3 culture medium containing the five control teeth (labeled 1–5) following incubation at 37 °C under culture-based microbiological conditions.

The culture tubes with studied samples were incubated at 37 °C for up to 10 days. The broth cultures were inspected regularly throughout the incubation period. Visible turbidity and/or sediment formation served as preliminary indicators of possible microbial growth. Representative culture tubes from the control group following incubation are shown in Figure 4. Samples demonstrating suspected growth, as well as samples without visible microbial growth, subsequently underwent microbiological confirmation by subculture onto appropriate solid media under aerobic and anaerobic conditions, followed by bacterial identification using matrix-assisted laser desorption/ionization time-of-flight mass spectrometry (MALDI-TOF MS) with the VITEK^®^ MS PRIME system (bioMérieux SA, Marcy-l’Étoile, France), as described in Section 2.7.

### 2.5. Dentin Processing Procedures

Dentin processing was performed strictly according to the manufacturers’ validated protocols, as previously described by Dłucik et al., to ensure standardized processing conditions and enable direct comparison of the microbiological performance of the three commercially available dentin-processing systems. No modifications were introduced to the recommended procedures.

The BM system involved mechanical grinding followed by chemical treatment with 3.5–5% hydrochloric acid (HCl), 70% ethanol, and 5% hydrogen peroxide (H_2_O_2_), with a total processing time of approximately 20 min. The TT system combined automated grinding, chemical decontamination, and partial demineralization using 25–50% hydrochloric acid (HCl), 10% hydrogen peroxide (H_2_O_2_), and demineralized water during an approximately 25 min processing cycle. The SDG system consisted of mechanical grinding followed by chemical treatment with 0.5 M sodium hydroxide (NaOH), 20% ethanol, and phosphate-buffered saline (PBS), with a total processing time ranging from 15 to 20 min. The chemical reagents used in each dentin-processing system are summarized in Table 2.

Following completion of each protocol, representative dentin samples were aseptically transferred into fresh Schaedler Broth K3 culture medium supplemented with vitamin K3 (bioMérieux, Marcy-l’Étoile, France). Post-processing microbiological assessment was initiated immediately using the same standardized culture-based methodology applied during baseline assessment to evaluate the presence of microbiologically detectable cultivable microorganisms following dentin processing.

### 2.6. UV Sterilization

A total of six pooled dentin samples, corresponding to the BM, TT, and SDG subgroups from storage groups 1 and 2, were additionally subjected to ultraviolet (UV) irradiation following completion of the dentin-processing protocols.

UV irradiation was performed using a Revolution UV sterilization unit (model 281239; Revolution, Robakowo, Poland), a commercially available device used for microbiological decontamination. The device operates at a power of 32 W, and dentin samples were exposed to UV irradiation for approximately 2 min 30 s, corresponding to the manufacturer’s standard operating cycle. The UV treatment was included as an exploratory supplementary decontamination procedure and was not considered a primary outcome of the present pilot study. Following UV irradiation, dentin samples were aseptically transferred into fresh Schaedler Broth K3 culture medium supplemented with vitamin K3 and incubated under the same conditions as those used during the baseline and post-processing culture-based microbiological assessments.

Because no cultivable microorganisms were detected following the standard dentin-processing protocols, no comparative analysis between UV-treated and non-UV-treated samples was performed. Accordingly, the UV procedure was not evaluated as an independent experimental variable in the present study.

### 2.7. Microbiological Analysis

Microbiological examinations were performed at the Department of Microbiology and Immunology, Medical University of Silesia in Katowice, Poland. Dentin samples and control teeth inoculated into Schaedler Broth K3 culture medium supplemented with vitamin K3 were incubated at 37 °C for up to 10 days under conditions suitable for the recovery of both aerobic and anaerobic microorganisms.

The broth cultures were inspected daily throughout the incubation period. Visible turbidity and/or sediment formation served as preliminary indicators of possible microbial growth. Samples demonstrating suspected growth, as well as all cultures at the completion of the 10-day incubation period, were subjected to further microbiological evaluation. A 20 μL aliquot of culture medium was inoculated onto solid culture media. Columbia agar supplemented with 5% sheep blood was used for aerobic cultivation, whereas Schaedler K3 agar was used for anaerobic cultivation. The inoculated media were incubated at 37 °C for 24–48 h under appropriate aerobic and anaerobic conditions. Microorganisms isolated from positive cultures were identified using matrix-assisted laser desorption/ionization time-of-flight mass spectrometry (MALDI-TOF MS) with the VITEK^®^ MS PRIME system (bioMérieux SA, Marcy-l’Étoile, France). The applied methodology was intended exclusively for culture-based microbiological assessment and was not designed to detect viable but non-culturable microorganisms, fungi, viruses, or bacterial endotoxins.

### 2.8. Statistical Analysis

Because the present investigation was designed as a pilot descriptive microbiological study, and only two positive microbiological cultures were observed, no formal inferential statistical analyses were performed. Furthermore, the use of pooled dentin samples (*n* = 24) as the microbiological unit of analysis precluded meaningful statistical comparisons among storage periods and dentin-processing systems. Accordingly, the results are presented descriptively as frequencies and percentages, and all interpretations are limited to the scope of this pilot culture-based microbiological investigation.

## 3. Results

### Microbiological Assessment

Microbiological assessment was performed on 24 pooled dentin samples representing eight storage periods and three dentin-processing systems, together with five freshly extracted control teeth.

Baseline culture-based microbiological assessment revealed detectable bacterial growth in 2 of 24 pooled dentin samples (8.3%). Positive cultures were identified in pooled samples representing the 2019 and 2023 storage groups. The isolated microorganisms were identified as *Staphylococcus epidermidis* and *Actinomyces viscosus*, respectively. Because dentin obtained from three teeth within each subgroup was pooled before microbiological analysis, it was not possible to determine which individual tooth was the source of contamination.

Following dentin processing, no microbiologically detectable bacterial growth by culture-based methods was observed in any pooled dentin sample processed using the BonMaker (BM), Tooth Transformer (TT), or Smart Dentin Grinder (SDG) systems. All processed dentin samples were considered culture-negative under both aerobic and anaerobic culture conditions using the culture-based methodology employed in the present study, irrespective of the dentin-processing protocol applied or the duration of refrigerated storage.

The absence of detectable microbial growth was confirmed in all processed dentin samples, including material prepared from teeth stored under refrigerated conditions for up to seven years. Similarly, no detectable microbial growth was observed in the six pooled dentin samples that additionally underwent ultraviolet (UV) irradiation following dentin processing.

Among the five control teeth, bacterial growth was detected in four samples (80.0%). The identified microorganisms included *Streptococcus anginosus* (two samples), *Streptococcus mutans* (one sample), and *Staphylococcus hominis* (one sample).

Overall, detectable bacterial growth was identified exclusively during baseline microbiological assessment, whereas all dentin samples remained culture-negative following processing, irrespective of storage duration or the dentin-processing system used. The distribution of microbiological findings is presented in Table 3, whereas the bacterial species identified by MALDI-TOF MS are summarized in Table 4.

## 4. Discussion

### 4.1. Clinical Relevance of Long-Term Tooth Storage and Principal Findings of the Study

The present study evaluated the microbiological status of dentin-derived biomaterials prepared from human teeth stored under refrigerated conditions for periods of up to seven years and subsequently processed using three commercially available chairside dentin-processing systems. Within the limitations of conventional culture-based microbiological methods, detectable bacterial contamination was identified only during baseline microbiological assessment, whereas all processed dentin samples remained culture-negative regardless of storage duration or the dentin-processing protocol applied. These findings provide preliminary microbiological evidence supporting the feasibility of long-term refrigerated tooth preservation under the investigated storage conditions as one component of the future multidisciplinary scientific validation of tooth banking.

The growing clinical interest in dentin-derived biomaterials has shifted the concept of tooth extraction from the disposal of biological waste toward the preservation of a patient-specific regenerative resource. In routine clinical practice, teeth are frequently extracted several months or years before regenerative procedures such as alveolar ridge preservation, sinus floor augmentation, or implant placement become necessary. Consequently, immediate chairside processing is not always feasible, and delayed clinical use requires reliable preservation strategies capable of maintaining the microbiological quality of stored teeth until processing. The present findings indicate that prolonged refrigerated storage did not compromise the microbiological effectiveness of the BM, TT, and SDG dentin-processing protocols under the investigated conditions.

The findings should be interpreted within the scope of the study design. The objective was not to establish the overall biological safety of long-term tooth preservation or to identify the optimal storage strategy for extracted teeth, but rather to determine whether microbiologically detectable bacterial contamination persisted following prolonged refrigerated storage and whether standardized dentin-processing protocols eliminated cultivable microorganisms before potential clinical use. Accordingly, the absence of detectable bacterial growth after processing should not be interpreted as definitive proof of biological or clinical safety.

From a broader perspective, the present investigation represents one component of the multidisciplinary scientific validation required for future tooth banking. Safe long-term preservation of extracted teeth requires multidisciplinary evidence extending beyond microbiological assessment. Together, these complementary scientific domains provide the foundation for future tooth banking.

### 4.2. Interpretation of the Microbiological Findings

The absence of cultivable microorganisms following dentin processing suggests that the BM, TT, and SDG protocols achieved effective microbiological decontamination under the investigated storage conditions. Within the limitations of conventional culture-based microbiological methods, these findings indicate comparable microbiological performance of the three commercially available dentin-processing systems, irrespective of storage duration.

The detection of *Staphylococcus epidermidis* and *Actinomyces viscosus* in two baseline pooled dentin samples is consistent with previous evidence indicating that viable microorganisms may persist within extracted teeth despite initial cleaning procedures and prolonged refrigerated storage. Bacteria may colonize dentinal tubules and survive within the complex dentin microstructure, where they are protected from mechanical surface cleaning alone [14,15,16,17,18]. The high frequency of positive cultures among freshly extracted control teeth further supports the ability of the applied microbiological protocol to recover viable oral microorganisms when present.

No association was observed between storage duration and the occurrence of detectable bacterial contamination. Positive baseline cultures were identified only in the 2019 and 2023 storage groups, whereas all remaining groups yielded culture-negative findings before dentin processing. Although the limited number of positive cultures precluded formal statistical analysis, these observations suggest that prolonged refrigerated storage was not associated with an increased frequency of cultivable bacterial contamination.

Comparable microbiological outcomes were obtained with the BM, TT, and SDG systems despite differences in grinding procedures, chemical decontamination protocols, and partial demineralization methods. These observations suggest that the evaluated processing protocols achieved similar microbiological effectiveness under the investigated conditions.

The absence of detectable bacterial growth does not constitute proof of complete sterility or biological safety but indicates that no cultivable microorganisms were recovered using the culture-based methods employed in this study. Conventional culture techniques remain the reference standard for detecting viable bacteria; however, they cannot exclude viable but non-culturable (VBNC) microorganisms, bacterial DNA, endotoxins, fungi, or viruses. Accordingly, complementary molecular, biological, clinical, and regulatory investigations will be required to comprehensively evaluate the long-term preservation of extracted human teeth for future regenerative applications.

### 4.3. Comparison with Previous Studies

The regenerative potential of autogenous dentin has been extensively documented in both experimental and clinical studies [3,4,5,6,7,8,9,10,11,12,13]. Previous investigations have primarily focused on the biological properties of dentin-derived biomaterials, clinical outcomes following bone augmentation, and the performance of commercially available dentin-processing systems.

Recent reviews support the clinical use of dentin-derived graft materials and emphasize the importance of standardized processing and decontamination protocols, which are considered essential for the safe clinical application of tooth-derived biomaterials [23,29,30,31]. Histological and clinical investigations have further demonstrated favorable regenerative outcomes following the use of different dentin-processing systems in bone augmentation procedures [8,32,33,34,35].

The present investigation extends previous studies by providing microbiological evidence for dentin biomaterials prepared from human teeth stored under refrigerated conditions for up to seven years. Although methodological differences preclude direct comparisons, our findings are consistent with previous reports supporting the microbiological effectiveness of standardized dentin-processing protocols [10,11,12,13,14,15,16,17,18].

### 4.4. Integration of Microbiological, Physicochemical, and Biological Evidence

Although the present study focused exclusively on microbiological assessment, these findings alone are insufficient to establish the scientific basis for long-term tooth preservation. Future clinical tooth banking requires complementary evidence demonstrating that prolonged storage preserves not only the microbiological quality but also the structural and biological properties of dentin-derived biomaterials.

Our recent Raman spectroscopy study demonstrated that dentin-derived biomaterials prepared from human teeth stored under identical refrigerated conditions for up to six years showed no significant deterioration of either the mineral or organic components of dentin, indicating preservation of physicochemical characteristics considered important for regenerative applications [27]. The present microbiological findings complement these observations by demonstrating comparable microbiological outcomes in dentin prepared from teeth stored for up to seven years. Together, these studies suggest that prolonged refrigerated storage did not adversely affect, under the investigated conditions, either the microbiological quality of processed dentin or the physicochemical integrity of the dentin matrix.

Nevertheless, microbiological and physicochemical evidence alone cannot determine the regenerative potential of dentin-derived biomaterials. Human dentin contains numerous bioactive molecules, including bone morphogenetic proteins (BMPs), transforming growth factor-β (TGF-β), insulin-like growth factors (IGFs), vascular endothelial growth factor (VEGF), and fibroblast growth factors (FGFs), all of which contribute to bone regeneration [3,4,5,6,7,8,9]. The present study did not evaluate preservation of these biological components following prolonged storage; therefore, no conclusions can be drawn regarding the biological activity of stored dentin.

Future investigations should determine whether prolonged storage influences the stability and biological function of dentin-derived growth factors using complementary molecular and immunohistochemical approaches. Together with clinical and regulatory evidence, these studies will provide the multidisciplinary scientific foundation required for the standardized preservation and future clinical reuse of extracted human teeth.

### 4.5. Toward an Evidence-Based Framework for Tooth Banking

The present study should be interpreted within the context of our broader research on dentin-derived biomaterials. Previous investigations have evaluated complementary aspects of tooth-derived grafts, including their clinical performance, histological characteristics, physicochemical stability, and, in the present study, microbiological quality. Together, these studies suggest that the scientific validation of tooth banking requires the integration of evidence from multiple complementary research domains rather than reliance on a single experimental endpoint.

Based on this multidisciplinary perspective, we propose the concept of Evidence-Based Tooth Banking (EBTB). Rather than representing a specific storage protocol or processing technology, EBTB is intended as a scientific framework integrating microbiological, physicochemical, biological, histological, clinical, and regulatory evidence to comprehensively evaluate preserved human teeth before consideration for future clinical application.

**Figure 5:**
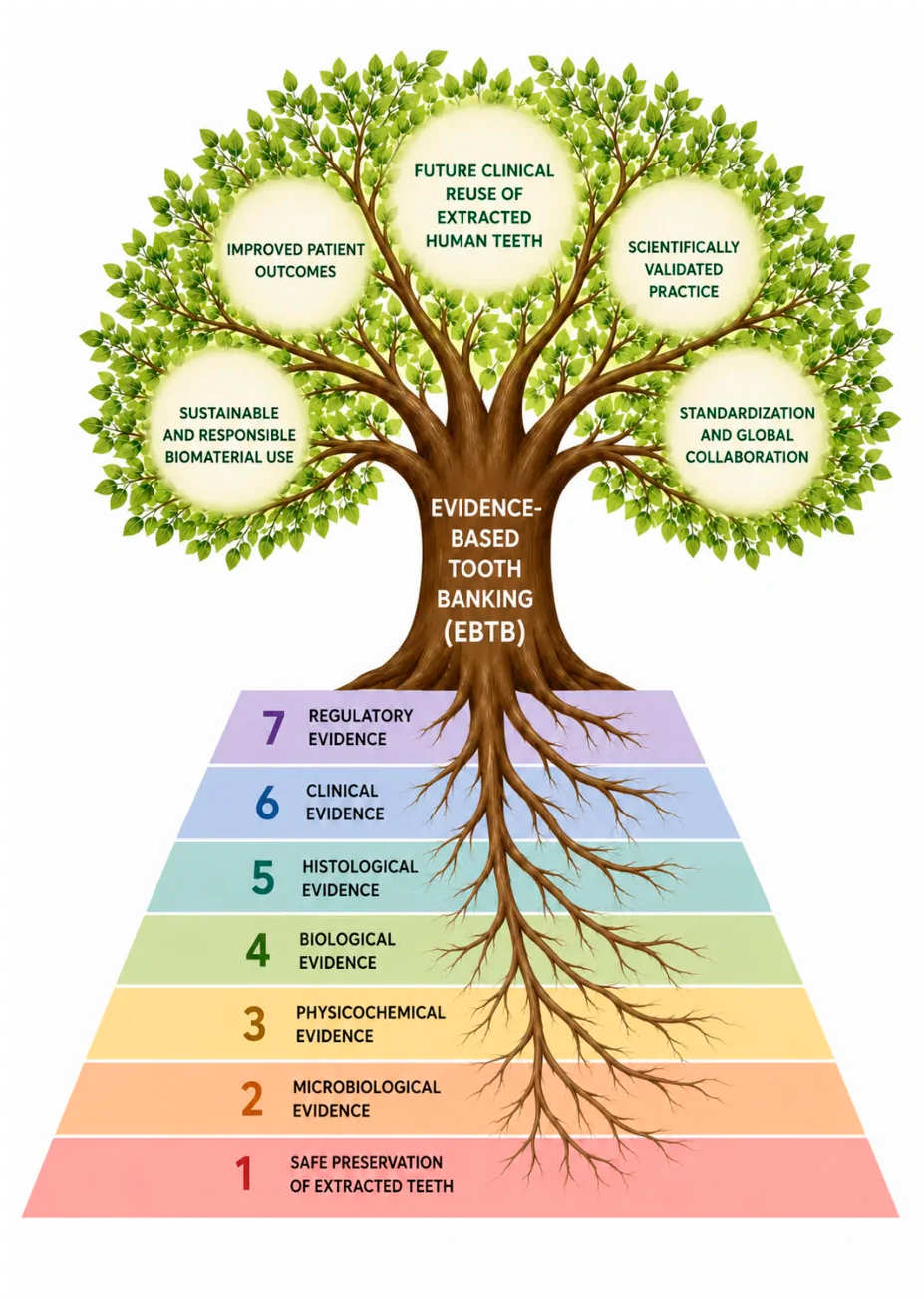
Conceptual model of Evidence-Based Tooth Banking (EBTB). The stepped foundation represents the complementary scientific evidence required for tooth banking, while the tree symbolizes their integration into a unified framework. Together, these domains support the standardized preservation and future clinical reuse of extracted human teeth.

The proposed framework is illustrated in Figure 5, which summarizes the complementary scientific domains contributing to tooth banking. Within this model, microbiological assessment represents one essential component that should be interpreted together with evidence from the remaining scientific domains. Accordingly, EBTB is proposed as a conceptual multidisciplinary research framework supporting the future scientific validation of tooth banking rather than an established clinical or regulatory standard. Future multidisciplinary investigations integrating complementary scientific evidence will be required to further develop, refine, and validate this framework before its potential clinical or regulatory implementation.

## 5. Limitations

The present study has several limitations that should be considered when interpreting the findings.

First, microbiological analyses were performed using pooled dentin samples obtained from three teeth within each experimental subgroup. Although pooling standardized the microbiological assessment and ensured sufficient material for culture analysis, it precluded identification of the individual tooth responsible for positive cultures and did not permit tooth-level statistical analyses.

Second, microbiological evaluation relied exclusively on conventional culture-based methods. Consequently, microorganisms in a viable but non-culturable (VBNC) state, fungi, viruses, and bacterial endotoxins could not be detected. Therefore, the absence of detectable microbial growth should not be interpreted as evidence of complete sterility or overall biological safety.

Third, this pilot study evaluated only one clinically applicable storage model based on long-term refrigerated preservation at approximately 4 °C. The objective was not to compare different storage strategies but to assess the microbiological status of dentin biomaterials prepared under routine storage conditions. Consequently, the findings cannot be generalized to other preservation methods, such as freezing, cryopreservation, or lyophilization.

Despite these limitations, the present study provides preliminary microbiological evidence supporting the feasibility of long-term refrigerated tooth preservation and contributes to the multidisciplinary validation required for the future clinical reuse of extracted human teeth.

## 6. Conclusions

Within the limitations of this pilot in vitro study, dentin biomaterials prepared from human teeth stored under long-term refrigerated conditions demonstrated no microbiologically detectable bacterial growth after processing with the BM, TT, and SDG systems. These findings provide preliminary culture-based microbiological evidence consistent with the microbiological effectiveness of standardized dentin-processing protocols under the investigated storage conditions.

Because this investigation was limited to qualitative culture-based microbiological assessment, the results should not be interpreted as evidence of complete sterility, overall biological safety, or clinical suitability of long-term preserved dentin biomaterials. Furthermore, the present study evaluated only one refrigerated storage protocol and therefore does not permit conclusions regarding the comparative effectiveness of alternative preservation strategies, including freezing, cryopreservation, or lyophilization.

Future multidisciplinary investigations integrating complementary scientific evidence are warranted to comprehensively evaluate long-term tooth preservation and to further develop, refine, and validate the proposed EBTB framework.

## Figures and Tables

**Table 1:** Distribution of Teeth Across Experimental Groups.

Group	Year of Extraction	BM (n)	TT (n)	SDG (n)	Control Teeth (n)	UV Irradiation
1	2018	3	3	3	—	Yes
2	2019	3	3	3	—	Yes
3	2020	3	3	3	—	No
4	2021	3	3	3	—	No
5	2022	3	3	3	—	No
6	2023	3	3	3	—	No
7	2024	3	3	3	—	No
8	2025	3	3	3	—	No
9 (Control Group)	2025	—	—	—	5	No
Total	—	24	24	24	5	—

BM—BonMaker; TT—Tooth Transformer; SDG—Smart Dentin Grinder.

**Table 2:** Dentin processing systems and corresponding chemical reagents used for dentin biomaterial preparation.

Device	Main Demineralizing Reagent	Additional Reagents	Processing Time
BonMaker (BM)	Hydrochloric acid (HCl), 3.5–5%	70% ethanol; 5% hydrogen peroxide	~20 min
Tooth Transformer (TT)	Hydrochloric acid (HCl), 25–50%	10% hydrogen peroxide; demineralized water	~25 min
Smart Dentin Grinder (SDG)	Sodium hydroxide (NaOH)	20% ethanol; phosphate-buffered saline (PBS)	15–20 min

Abbreviations: HCl, hydrochloric acid; NaOH, sodium hydroxide; PBS, phosphate-buffered saline.

**Table 3:** Microbiological findings before and after dentin processing. Data are presented as number of samples and percentages.

Sample Category	Positive Cultures, n (%)	Negative Cultures, n (%)
Baseline pooled dentin samples (n = 24)	2 (8.3)	22 (91.7)
Control teeth (n = 5)	4 (80.0)	1 (20.0)
BonMaker (BM) after processing (n = 8)	0 (0)	8 (100)
Tooth Transformer (TT) after processing (n = 8)	0 (0)	8 (100)
Smart Dentin Grinder (SDG) after processing (n = 8)	0 (0)	8 (100)

**Table 4:** Microorganisms identified during microbiological assessment.

Sample Source	Identified Microorganism
Pooled dentin sample representing the 2019 storage group	*Staphylococcus epidermidis*
Pooled dentin sample representing the 2023 storage group	*Actinomyces viscosus*
Control tooth 1	*Streptococcus anginosus*
Control tooth 3	*Staphylococcus hominis*
Control tooth 4	*Streptococcus mutans*
Control tooth 5	*Streptococcus anginosus*

## Data Availability

The datasets generated and analyzed during the current study are available from the corresponding author upon reasonable request.
